# Interval between two-stage exchanges: what is optimal and how do you know?

**DOI:** 10.1186/s42836-023-00185-4

**Published:** 2023-07-05

**Authors:** Ricardo Sousa, André Carvalho, Daniel Soares, Miguel Araújo Abreu

**Affiliations:** 1 Department of Orthopedics, Centro Hospitalar Universitário de Santo António, 4099-001 Porto, Portugal; 2 Porto Bone and Joint Infection Group (GRIP), Centro Hospitalar Universitário de Santo António and CUF Hospitais E Clínicas, 4099-001 Porto, Portugal; 3 Department of Infectious Diseases, Centro Hospitalar Universitário de Santo António, 4099-001 Porto, Portugal

**Keywords:** Periprosthetic joint infection, Two-stage exchange arthroplasty, Reimplantation timing, Serological markers, Antibiotic holiday, Cement spacer aspiration

## Abstract

**Background:**

Two-stage exchange arthroplasty remains the most popular option for the treatment of chronic periprosthetic joint infection (PJI). Determining infection eradication and optimal timing of reimplantation can be challenging. Information to allow for a truly informed evidence-based decision is scarce.

**Methods:**

We conducted a critical review of available evidence on the presently available tests to help determine timing of reimplantation.

**Results:**

Serology is traditionally used to follow up patients after the first stage. Despite tradition mandates waiting for normal inflammatory markers, there is actually no evidence that they correlate with persistent infection. The role of synovial fluid investigation between stages is also explored. Cultures lack sensitivity and neither differential leukocyte counts nor alternative biomarkers have proven to be accurate in identifying persistent infection with a spacer in situ. We also examined the evidence regarding the optimal time interval between resection and reimplantation and whether there is evidence to support the implementation of a two week “antibiotic holiday” prior to proceeding with reimplantation. Finally, wound healing and other important factors in this setting will be discussed*.*

**Conclusion:**

Currently there are no accurate metrics to aid in the decision on the optimal timing for reimplantation. Decision must therefore rely on the resolution of clinical signs and down trending serological and synovial markers.

## Introduction

Periprosthetic joint infection (PJI) is the archetypal biofilm-related infection. As such, once a mature biofilm has developed onto the implant surface, complete removal of the infected prosthesis, devitalized bone and periprosthetic soft tissues is the only way to ensure biofilm eradication [1].

While single-stage revision arthroplasty is gaining momentum, especially in selected cases, a two-stage approach remains the gold standard treatment modality for chronic PJI [2–4].

The two-stage approach consists of an initial surgery where a thorough debridement and removal of implants is performed. Concurrently, multiple samples for microbiology and histology are taken and an antibiotic loaded spacer is temporarily inserted. Subsequently, patients are administered 6 weeks of intravenous antibiotics and when the infection is deemed eradicated, the patient undergoes a second procedure. The second stage involves removing the spacer, further debridement, and reimplantation of components.

To date, we are yet to identify any markers that can determine timing of reimplantation [5]. The purpose of this study is to perform a critical review of the available evidence regarding decision-making on the optimal timing to proceed with the second stage.

## Serological testing

Serum inflammatory markers, such as erythrocyte sedimentation rate (ESR) and C-reactive protein (CRP), are extensively studied in the diagnostic approach, culminating in their inclusion in several diagnostic criteria [6, 7]. In addition to this, they are widely used in clinical practice to monitor infection eradication. Classically, a downward trend of both markers has been used to determine eradication of infection.

Multiple studies throughout the literature have not been able to find accurate thresholds to predict persistence of infection as they frequently remain elevated despite proven infection eradication [8–10]. In fact, it has been estimated that normal inflammatory markers have a low sensitivity of around 50%, and slightly better specificity of about 70% [11, 12]. Even if you try to analyze the variation in serological values between stages, not only absolute values before the second stage, it seems there is no additional diagnostic accuracy [13, 14].

Coagulation-related biomarkers, such as D-dimer and fibrinogen may be possible alternatives of interest in the diagnosis of PJI [15, 16]. They have also been studied as predictors in determining the optimal timing of reimplantation, but with conflicting results. Despite the initial promise [17], further studies focusing on D-dimer have shown it to be unreliable to accurately predict persistence of infection between stages [18, 19]. Similar conflicting results can be found on the role of fibrinogen in these circumstances [14, 20]. A possible role for plasma Interleukin-6 in two-stage revision arthroplasty may also exist but more studies are needed due to the contradictory findings of the available evidence [14, 21, 22].

Although no specific marker or threshold is available as a sole guide to decision-making, a downward trend (not necessarily complete normalization), specifically of CRP, seems to be of good prognostic value [23, 24].

## Synovial fluid investigation

Synovial fluid analysis has long been recognized for having excellent accuracy in the diagnosis of PJI. Once fluid is collected from the affected joint, it can be used to perform several tests. Although the role of synovial fluid analysis is of undisputable importance in the diagnosis of PJI in patients presenting with a painful prosthesis, its role in determining the optimal timing of reimplantation remains unclear.

### Cultures

Although the value of synovial fluid culture is indisputable in the preoperative workup of suspected PJI, many studies have demonstrated the that culture of the aspirated fluid failed to provide accurate diagnosis of PJI, and the sensitivity was especially low [25–28]. This relates to the pathophysiology of biofilm-related infections of implants. Most bacteria are present in the implant itself and in neighboring periprosthetic tissues and not many planktonic bacteria are found in the synovial fluid.

In the context after the first-stage surgery, this is further aggravated by the systemic antibiotic therapy and existing antibiotics in the spacer itself. It is well-established that cultures of synovial fluid aspiration between stages have consistently shown poor sensitivity, even after a two-week “antibiotic holiday”. Although some researchers, such as Preininger et al. [29] and Macke et al. [30] did show somewhat higher sensitivity, at 21% and 57%, respectively, most studies found the sensitivity to be extremely low, ranging from 0% to 6% [12, 31–34]. On the other hand, specificity was much higher, ranging from 85%–99%. However, false positives resulting in this setting remain a concern [12, 29–35].

### Leukocyte count

Differential cell count is the best studied biomarker for the diagnosis of PJI. A number of different optimal thresholds have been proposed over the years and different PJI definitions use different values for interpretation [6, 7, 36].

Furthermore, the utility of synovial fluid cell count in determining timing of reimplantation has also been examined with different cutoffs, ranging from just under 1,000 to over 8,200 cells/μL for white blood cell count and 52%–80% for PMN% [9, 10, 12, 31–33, 37–39]. Even considering each study’s ideal threshold, both total leukocyte count and proportion of PMN offer suboptimal diagnostic accuracy. Total leukocyte count sensitivity ranges from 10%–50% in some studies [12, 31–33, 38–40] to 75%–82% in others [9, 10, 37]. Specificity was somewhat higher with most studies, placing it around 70%–97% [9, 12, 31, 32, 37–39] although some offered lower accuracy [10, 33]. Proportion of PMN followed a similar pattern [9, 10, 12, 37–39] (See Table 1).

**Table 1:** Summary findings on differential leukocyte count in identifying persistent infection in patients with spacers

Publication	Population	Proposed cutoff	Sensitivity	Specificity	Positive predictive value	Negative predictive value	Accuracy
Shukla et al. 2010 [9]	87 hip spacers	3,528 cells/μL	78%	96%	-	-	94%
		79% PMN	78%	82%	-	-	82%
Kusuma et al. 2011 [10]	76 knee spacers	1,102 cells/μL	75%	61%	-	-	62%
		71% PMN	75%	66%	-	-	66%
Hoell et al. 2016 [33]	115 spacers (56 hips and 59 knees)	970 cells/μL	31%	39%	11%	71%	-
		-	-	-	-	-	-
Newman et al. 2017 [38]	77 hip spacers	3,000 cells/μL	47%	87%	50%	85%	78%
		80% PMN	76%	80%	52%	92%	79%
Zmistowski et al. 2017 [39]	128 spacers (40 hips and 88 knees)	1,234 cells/μL	44%	77%	26%	88%	70%
		57% PMN	67%	59%	24%	90%	60%
Muhlhofer et al. 2018 [12]	141 spacers (45 hips and 68 knees)	Not specified	10%	81%	10%	81%	-
		Not specified	10%	79%	9%	81%	-
Boelch et al. 2018 [31]	94 knee spacers	4,450 cells/μL	50%	76%	17%	97%	-
		-	-	-	-	-	-
Boelch et al. 2018 [32]	92 hip spacers	2,000 cells/μL	25%	97%	67%	82%	-
		-	-	-	-	-	-
Ascione et al. 2021 [37]	82 knee spacers	934 cells/μL	82%	82%	41%	98%	-
		52% PMN	82%	78%	36%	97%	-

The use of total leukocyte count and proportion of polymorphonuclear neutrophils (PMN) to detect persistence of infection before reimplantation is even more complicated by the fact that there is no gold standard definition of what constitutes a persistent infection. The vast majority of studies used positive cultures at the reimplantation stage as the benchmark, but negative cultures are not a guarantee of infection eradication [39, 40]. More recently, Pannu et al. [41] have recently suggested that elevated differential cell count might predict treatment outcomes at a minimum of 1-year follow-up.

### Biomarkers

There are a number of alternative biomarkers currently recognized as valuable for PJI diagnosis [42]. Attempting to overcome the aforementioned limitations, a number of them have also been considered before second stage revision surgery.

Apart from differential cell count, alpha-defensin (AD) is the most widely studied biomarker. Using the 2018 ICM criteria [43] to define persistent infection during the second stage, Stone et al*.* [44] found the sensitivity and specificity of the AD test resulted in diagnosing PJI in a cohort of 52 procedures to be 71% and 98%, respectively. Nevertheless, subsequent studies did not confirm these promising results. More recently, Owens et al. [45] enrolled patients undergoing reimplantation with AD testing preoperatively. Fifteen cases were diagnosed as “not infected” and none of them had positive cultures or a positive AD. The majority of them (*n* = 68) were classified as “possibly infected”. In this group, 67 patients had a negative AD test (98.5%) and 1 patient had an “indeterminate” AD test. Furthermore, four cases were classified as “infected” and none had a positive AD test or positive cultures. They concluded that routine use of AD in the work-up prior to a second-stage procedure for PJI is not warranted. A different approach looking at the Delphi criteria [46] for persistent PJI at 1 year follow-up was pursued by other authors. Samuel et al*.* [47] examined AD results prior to reimplantation and found that it had poor sensitivity (7%) and poor overall accuracy (73%). Bielefeld et al*.* [40] studied a cohort of 20 patients and also found limited sensitivity (33%) and specificity (53%).

Leukocyte esterase (LE) is a simple and inexpensive test and has demonstrated excellent accuracy for the diagnosis of PJI [42]. Nonetheless, there are scarce data on its performance in identifying persistent infection between stages. Kheir et al. [48] gathered the results of 77 patients with a minimum of 90-day follow-up to assess treatment failure, as defined by the Delphi criteria (18/95 patients of the original cohort were excluded due to blood contamination of LE test strip). The LE test was positive in 26% (5/19) of the patients with persistence of infection and/or subsequent failure and was negative in all the patients who had not failed at the latest follow-up. It yielded a limited sensitivity of 26%. The same trend was found in the previously mentioned study by Bielefeld et al. [40], where the LE test yielded a sensitivity of 0% by failing to identify any of the three reinfection cases (See Table 2).

**Table 2:** Summary findings on the performance of synovial fluid biomarkers in identifying persistent infection in patients with spacers

Publication	Population	Studied biomarker (cutoff)	Sensitivity	Specificity	Positive predictive value	Negative predictive value	Accuracy
Frangiamore et al. 2016 [49]	32 spacers	IL-6 (8.7 pg/mL)	0%	89%	0%	86%	78%
Kheir et al. 2017 [48]	77 spacers	Leukocyte esterase (+ 2)	26%	100%	100%	87%	63%
Stone et al. 2019 [44]	52 spacers (22 hips and 30 knees)	Alpha-defensin	71%	98%	83%	96%	-
Samuel et al. 2019 [47]	69 spacers (26 hips and 43 knees)	Alpha-defensin	7%	89%	14%	79%	83%
Bielefeld et al. 2021 [40]	20 spacers (8 hips and 12 knees)	Alpha-defensin (4.8 ng/mL)	33%	53%	-	-	41%
		Leukocyte esterase (+ 2)	0%	100%	-	-	-
Owens et al. 2022 [45]	87 spacers (27 hips and 60 knees)	Alpha-defensin	6%^a^	100%	-	94%	83%

^a^If you consider one undeterminate AD test result in an infected case as not negative

Other biomarkers for the diagnosis of PJI are also available but information about their performance in this specific context is scarce. Frangiamore et al. [49] looked at a number of cytokines and found that synovial interleukin-6 had the highest overall accuracy (78%), and a 0% sensitivity for detecting treatment failure. In the study by Wouthuyzen-Bakker et al*.* [50], while validating calprotectin for PJI diagnosis, 9 patients in the control group with a spacer in situ and with infection deemed to be cured, yielding promising results that naturally require more studies.

## Antibiotic holiday period

After the first stage surgery and once the planned antibiotic period is over, an antibiotic-free time interval, also known as a drug holiday, was classically encouraged. The rationale is to allow a latent persistent infection to manifest before the second-stage. After the drug holiday, the patient underwent clinical and laboratory re-evaluation before deciding whether to proceed with reimplantation. This recommendation remains controversial.

Bejon et al. [51] retrospectively reported on 152 patients, 12% of whom were not preceded by a 2-week antibiotic-free period before reimplantation. Positive microbiology at reimplantation was not significantly different in patients without the holiday period when compared to those operated 2-weeks after antibiotic discontinuation (16% (3/18) vs. 13% (18/134)). Furthermore, the vast majority of unplanned debridement following the first stage were carried out before antibiotics were stopped (25 vs. 2 procedures). Tan et al*.* [52] analyzed a large multicentre retrospective cohort of 785 PJIs treated with a two-stage approach. When they looked at the 409 cases that were reimplanted with no surgeries in the interim stage, the average duration of the antibiotic holiday period lasted 30 days, with 9.5% (*n* = 39) having less than a 1-week period, 19.6% (*n* = 80) having less than a 2-week period, and 42.5% (*n* = 174) having less than a 4-week antibiotic holiday period. There was no difference in the treatment failure rate between them. However, when looking at patients receiving unplanned surgeries performed before reimplantation for persistent or recurrent infection, they found that most of them (55/94) failed during the antibiotic holiday period, at a mean of 26 days after discontinuation of antibiotics. Some authors have recommended against antibiotic discontinuation for many years now [53] and perform the second stage under antibiotic therapy that is maintained for a total of at least 12 weeks, regardless of the duration of the time interval with the spacer. This approach has consistently shown good outcomes [54, 55]. Ascione et al. [56] directly compared both strategies in a total of 196 patients with PJI treated with a two-stage protocol. There were 114 patients treated with continuous antibiotic therapy and 8 of them had positive microbiologic findings at reimplantation. Eighty-two patients experienced a 15-day (median) antibiotic-free period before reimplantation, and 9 of them had positive cultures. More importantly, outcomes after reimplantation were significantly better in patients treated with continuous antibiotic therapy (91% (104/114) vs. 79% (65/82)).

## Time to reimplantation

It follows, from what was previously discussed, that the proper timing for second-stage revision surgery is also a matter of great debate. Multiple studies reported that time to reimplantation ranged from a few weeks to several months or even longer [57]. This heterogeneity stems from the common belief that a delayed second stage will result in higher rate of treatment success. The rationale is that if infection does not emerge after a prolonged period of time one can be more certain that it is eradicated. However, there is no evidence to support this claim.

Unless the wound was slow to heal or there was extensive bone destruction, Haddad et al. [58] reported no increase in reinfection rates by reducing the interval to 3 weeks. Several other papers have also demonstrated that it is possible to achieve good results with short intervals of 2–4 weeks provided that there are favorable bone and soft tissue conditions and in the absence of drug resistant microorganisms [53–55, 59]. Furthermore, a number of studies have even shown an increased re-infection risk associated with prolonged time interval between stages [60–64]. Fu et al. [60] followed 81 two-stage total knee PJI who underwent two-stage revision and found only three failures out of 40 patients between 12 and 16 weeks and seven out of 41 reimplantations with a more than 16-week interval. Aali Rezaie et al. [61] retrospectively looked at 282 patients with an average time to reimplantation of 100 days. They found that patients reimplanted at > 26 weeks were twice as likely to fail in comparison to those reimplanted within < 26 weeks (43.8% vs. 21.1%). Vielgut et al*.* [62], in a study of 77 patients, determined that the optimal spacer retention time should be less than 12 weeks. They found a sixfold higher risk of getting a reinfection in the 35 patients with a prolonged spacer retention period (31.4%) compared to the 39 patients with an optimal spacer retention period (7.7%). This trend was further confirmed by Borsinger et al. [63] who looked at 101 cases and stratified spacer interval time into < 12 weeks, 12–18 weeks and > 18 weeks. Time to reimplantation longer than 18 weeks was associated with higher rates of treatment failure at 2 years in a multivariable analysis accounting for other variables such as prior revision or ASA score. What is not entirely clear from these retrospective studies is the exact reason(s) why certain patients were left longer with the spacer. One can only hypothesize whether they had other unfavorable conditions such as poor soft tissues, more significant comorbidities or patients being managed by less experienced surgeons, etc.

Still, in addition to the reinfection risk, prolonged spacer time is associated is associated with worse clinical and functional results [65, 66].

## Favorable wound healing and other major factors

Unhealthy soft tissues around the affected joint are a constant source of concern and a frequent limitation/restraint to the choice of surgical treatment in PJI. They can further be disturbed by the initial debridement performed on the first stage. Poor soft tissue conditions may compromise effective wound closure, giving rise to prolonged wound drainage and delayed wound healing that are well-established risk factors for infection [67] or even resulting in joint exposure which needs flap coverage.

**Fig. 1:**
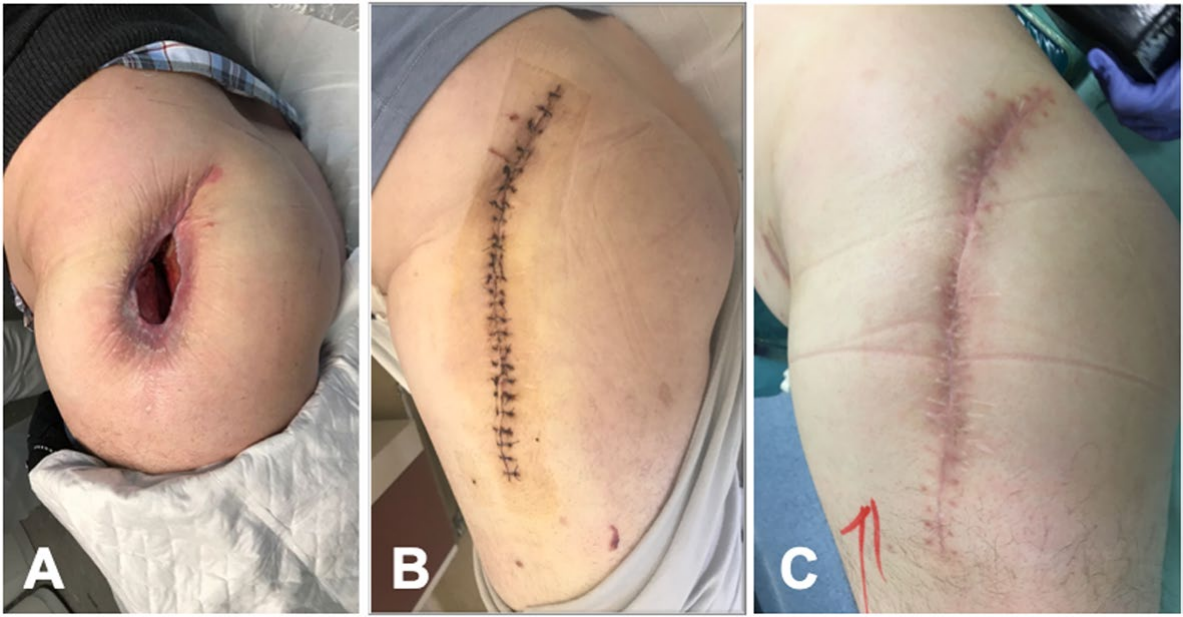
Left hip: preoperative clinical aspect (**A**); three weeks after operation (**B**); and 6 weeks after operation with uneventful favourable healing (**C**)

**Fig. 2:**
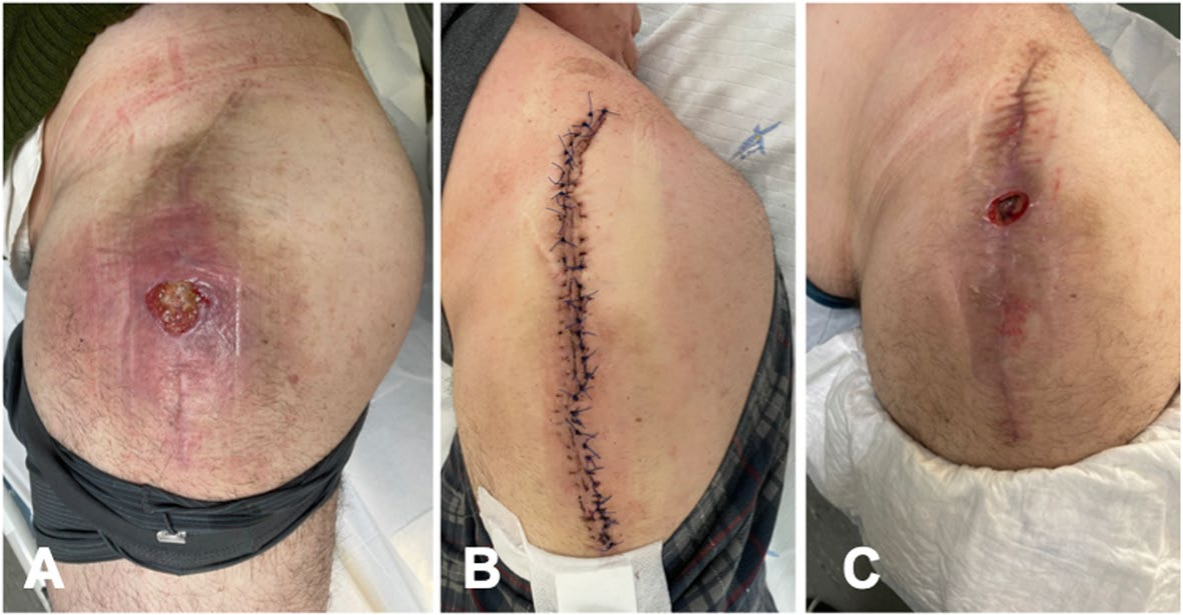
Left hip: preoperative clinical aspect (**A**); three weeks after operation (**B**); and 6 weeks after operation with wound failure suggesting persistent infection despite normal inflammatory markers (**C**)

**Fig. 3:**
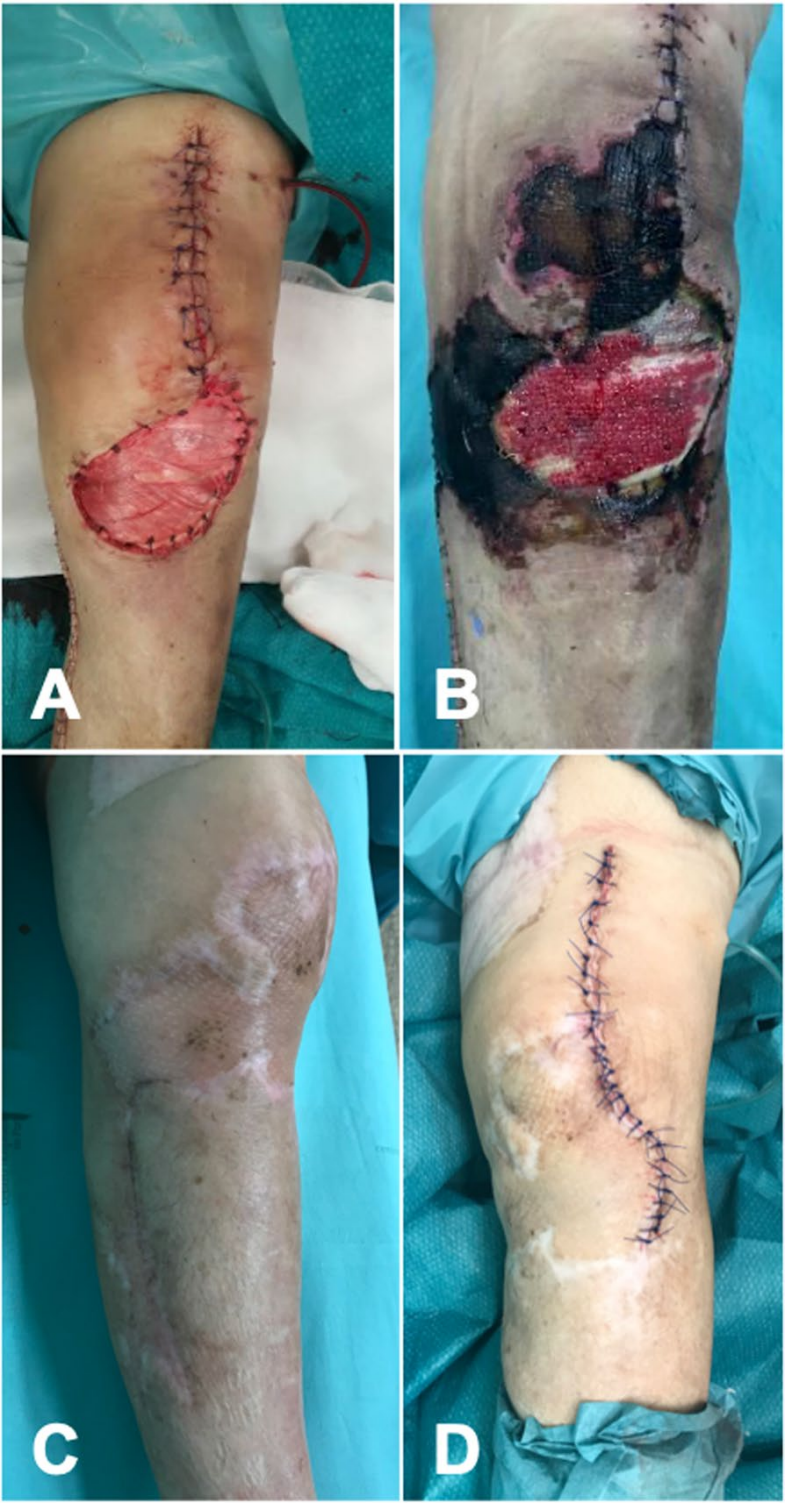
Clinical aspect immediately after the first-stage left knee surgery with medial gastrocnemius flap (**A**); three weeks after first stage with additional extensive skin necrosis (**B**); clinical aspect before the second stage almost one-year after initial surgery (**C**); immediate post operation after the second stage leading to uneventful healing (**D**)

**Fig. 4:**
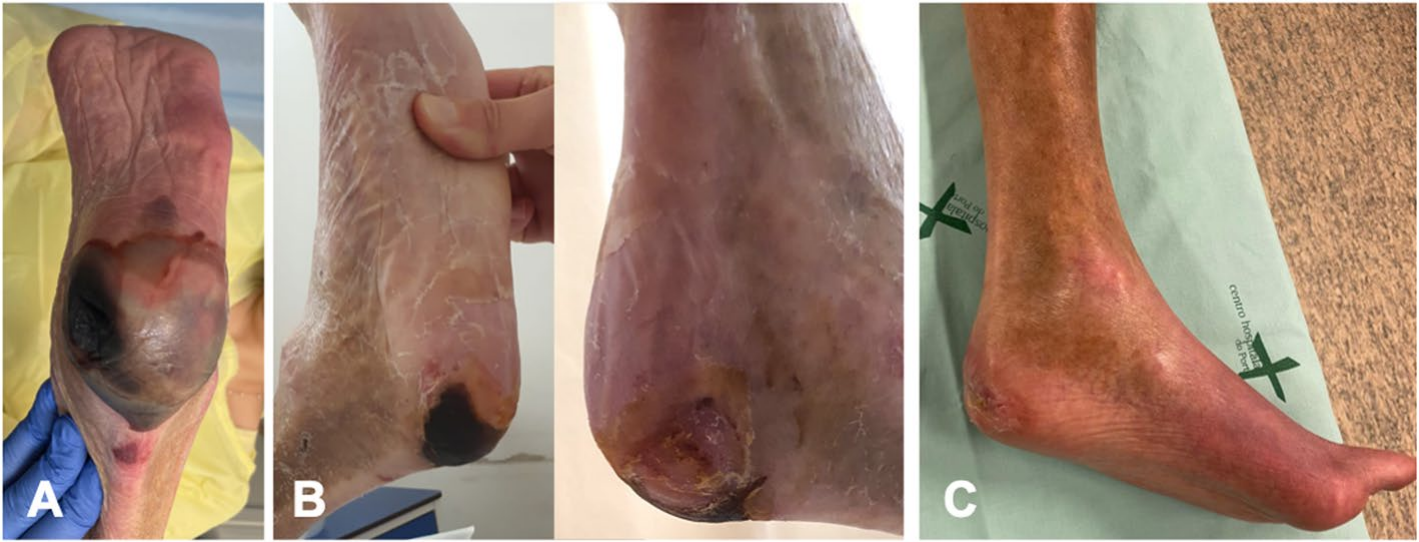
Extensive calcaneus skin necrosis in a diabetic patient at the time of left hip PJI first stage (**A**); six weeks after first stage, i.e., two weeks after revascularization (**B**); at the time of the second stage with complete ulcer healing after six months (**C**)

It is therefore only natural that appropriate wound healing is a critical part of deciding whether to proceed with reimplantation. Of note, clinical evaluation is extremely helpful and should never be overlooked. In addition to this, uneventful wound healing and the lack of inflammatory signs are good predictors, even if serum inflammatory markers are not completely normal (Fig. 1). On the other hand, even “minor” wound healing problems are signs of infection persistence, even if inflammatory markers are negative (Fig. 2). In addition, it might be necessary to wait much longer than the “normal” few weeks between the first and second stage if skin complications occur and there is the need for extensive soft tissues healing (Fig. 3). Addressing general health status and optimizing medical comorbidities such as malnutrition, or diabetes is also an important aspect of managing patients in the interval between stages (Fig. 4).

## Persistent infection and subsequent outcomes

If persistence of infection is suspected, further debridement and spacer exchange rather than definitive reconstruction is warranted. Furthermore, it is well established that positive cultures during reimplantation are associated with increased risk of reinfection [68–71]. Notwithstanding, we are yet to reach a consensus on what constitutes a persistent infection. Criteria (other than microbiological) originally intended for the diagnosis of PJI are often used as a standard but the biological environment after the first stage is clearly different. The first stage elicits a physiological inflammatory response that hampers interpretation of serological and synovial parameters and biomarkers. Thus, it is not surprising that such criteria have low accuracy for detecting successful or failed treatment following reimplantation [47–49].

## Conclusion

The main purpose of the interval between two-stage exchanges is to eradicate infection prior to reimplantation. Identifying persistence of infection before reimplantation is therefore of paramount importance. Unfortunately, we are currently unable to identify any metrics or test(s) to accurately identify persistence of infection prior to proceeding with reimplantation. In addition to this, the interval between stages is also important to maximizing chance of successful outcomes. The decision to proceed with reimplantation must therefore take into consideration the following major aspects. Firstly, appreciating that the original infection is cured must rely on a combination of clinical evaluation of the joint (uneventful wound healing, no inflammatory signs, etc.) and down-trending serological markers (not necessarily complete normalization). Furthermore, joint aspiration is not routinely recommended but may be useful in cases of uncertainty. Still, one should be aware of the lack of sensitivity of the standard diagnostic procedures. Secondly, it is important to ensure the best possible local and systemic circumstances to minimize the risk of PJI associated with revision arthroplasty. This often means waiting for appropriate local soft tissue conditions, and, whenever possible, eradication of possible foci of infection elsewhere.

## Data Availability

No datasets were generated or analyzed in this paper. The referenced data are part of clinical study publications listed in the bibliography.

## Abbreviations

PJI: Periprosthetic Joint Infection
ESR: Erythrocyte Sedimentation Rate
CRP: C-reactive Protein
PMN: Polymorphonuclear Neutrophils
AD: Alpha-defensin
LE: Leukocyte Esterase
